# Supplementary material for: Correlation of the disease-specific Canadian Cardiovascular Society (CCS) classification and health-related quality of life (15D) in coronary artery disease patients
Source: PLoS One. 2022 Apr 1;17(4):e0266101. doi: 10.1371/journal.pone.0266101 (PMC8975144; doi:10.1371/journal.pone.0266101)
Supplement: S1 Table — (PDF) [file pone.0266101.s001.pdf]

S1 Table. Spearman correlation coefficients (95% confidence interval) between change in the CCS and the 15D in different subgroups.

Subgroup	N	Sample Correlation	95% Confidence Interval	
**All patients**	805	0.33	0.27	0.40
OMT	362	0.27	0.18	0.37
CABG	168	0.29	0.15	0.43
PCI	275	0.39	0.28	0.48
Male	529	0.39	0.31	0.46
Female	276	0.22	0.11	0.33
<70	425	0.37	0.29	0.45
≥70	380	0.28	0.18	0.37
