# Supplementary material for: Correlation of the disease-specific Canadian Cardiovascular Society (CCS) classification and health-related quality of life (15D) in coronary artery disease patients
Source: PLoS One. 2022 Apr 1;17(4):e0266101. doi: 10.1371/journal.pone.0266101 (PMC8975144; doi:10.1371/journal.pone.0266101)
Supplement: S2 Table — (PDF) [file pone.0266101.s002.pdf]

S2 Table. Spearman correlation coefficients (95% confidence interval) between change in the CCS and the 15D's dimensions.

15D dimension	N	Sample Correlation	95% Confidence Interval	
Breathing	810	0.40	0.34	0.45
Depression	811	0.14	0.07	0.21
Discomfort and symptoms	809	0.15	0.08	0.21
Distress	808	0.10	0.03	0.17
Eating	812	0.04	-0.03	0.11
Excretion	811	0.05	-0.01	0.12
Hearing	811	0.07	0.00	0.14
Mental function	811	0.08	0.01	0.15
Mobility	813	0.16	0.09	0.22
Vision	809	0.11	0.04	0.18
Sexual activity	808	0.12	0.06	0.19
Sleeping	810	0.09	0.02	0.15
Speech	811	0.05	-0.02	0.11
Usual activities	811	0.19	0.12	0.25
Vitality	812	0.30	0.24	0.36
