# Supplementary material for: Correlation of the disease-specific Canadian Cardiovascular Society (CCS) classification and health-related quality of life (15D) in coronary artery disease patients
Source: PLoS One. 2022 Apr 1;17(4):e0266101. doi: 10.1371/journal.pone.0266101 (PMC8975144; doi:10.1371/journal.pone.0266101)
Supplement: Attachment — Submitted filename: Response to reviewers.docx [file pone.0266101.s005.docx]

**PONE-D-21-19920R1**

**Correlation of the disease-specific Canadian Cardiovascular Society (CCS) classification and health-related quality of life (15D) in coronary artery disease patients**

**Response to Reviewers:**

1- The authors made several corrections and as the manuscript was sent for a new evaluation, there is no way to evaluate it, because the corrections and the first version of the manuscript are together in the same version, the correction with marking (ie: ~~manuscript~~), leaving it confusing for the correction.

I suggest a new submission, only with the second version of the manuscript and that the authors highlight what was changed (i.e: **manuscript**).

**Our response:** *We apologize for the inconvenience. However, the revised manuscript is submitted with tracked changes (utilizing “Track changes” tool in Microsoft Word) as suggested by the journal. In addition, the submission includes a clean version without tracked changes.*

2- I also suggest that authors review the order of references in the introduction. The paragraphs were redacted, but the reference number did not change.

**Our response**: *We are grateful for the Reviewer for noticing this. We have reviewed and corrected the order of the references.*
